# Small GTPases in platelet membrane trafficking

**DOI:** 10.1080/09537104.2018.1535703

**Published:** 2018-10-26

**Authors:** Tony G. Walsh, Yong Li, Andreas Wersäll, Alastair W. Poole

**Affiliations:** From the School of Physiology, Pharmacology and Neuroscience, Biomedical Sciences Building, University of Bristol, Bristol, UK

**Keywords:** Endocytosis, exocytosis, GTPase, platelets, trafficking

## Abstract

Our understanding of fundamental biological processes within platelets is continually evolving. A critical feature of platelet biology relates to the intricate uptake, packaging and release of bioactive cargo from storage vesicles, essential in mediating a range of classical (haemostasis/thrombosis) and non-classical (regeneration/inflammation/metastasis) roles platelets assume. Pivotal to the molecular control of these vesicle trafficking events are the small GTPases of the Ras superfamily, which function as spatially distinct, molecular switches controlling essential cellular processes. Herein, we specifically focus on members of the Rab, Arf and Ras subfamilies, which comprise over 130 members and platelet proteomic datasets suggest that more than half of these are expressed in human platelets. We provide an update of current literature relating to trafficking roles for these GTPases in platelets, particularly regarding endocytic and exocytic events, but also vesicle biogenesis and provide speculative argument for roles that other related GTPases and regulatory proteins may adopt in platelets. Advances in our understanding of small GTPase function in the anucleate platelet has been hampered by the lack of specific molecular tools, but it is anticipated that this will be greatly accelerated in the years ahead and will be crucial to the identification of novel therapeutic targets controlling different platelet processes.

## Introduction

Membrane trafficking describes a form of cellular communication involving the movement of cargo (e.g. proteins, lipids or pathogens), within membrane bound vesicles, towards spatially distinct compartments. This is a multistep process involving initial vesicle formation from a donor organelle membrane where cargo is packaged (e.g. *trans*-Golgi network), movement of this ‘carrier’ vesicle towards an acceptor organelle (e.g. early endosome) and, tether to an acceptor with subsequent membrane fusion allowing release of carrier cargo into the lumen of the target organelle [1]. Classically, two of the principal roles for membrane trafficking are exocytosis and endocytosis, which terminate or initiate at the plasma membrane, respectively. At the heart of these processes is the biosynthetic pathway primarily composed of the endoplasmic reticulum (ER) and Golgi complexes to orchestrate the sorting of correctly folded proteins into vesicles, prior to trafficking to a target organelle. These complex and tightly regulated processes are fundamental to eukaryotic cell function, and as such, the Nobel Prize in Physiology or Medicine 2013 was awarded to three pioneering scientists (Rothman, Schekman & Sϋdhof) who discovered key molecular regulators of vesicular traffic in mammalian cells [2].

## Membrane Trafficking in Platelets

**Figure 1.**
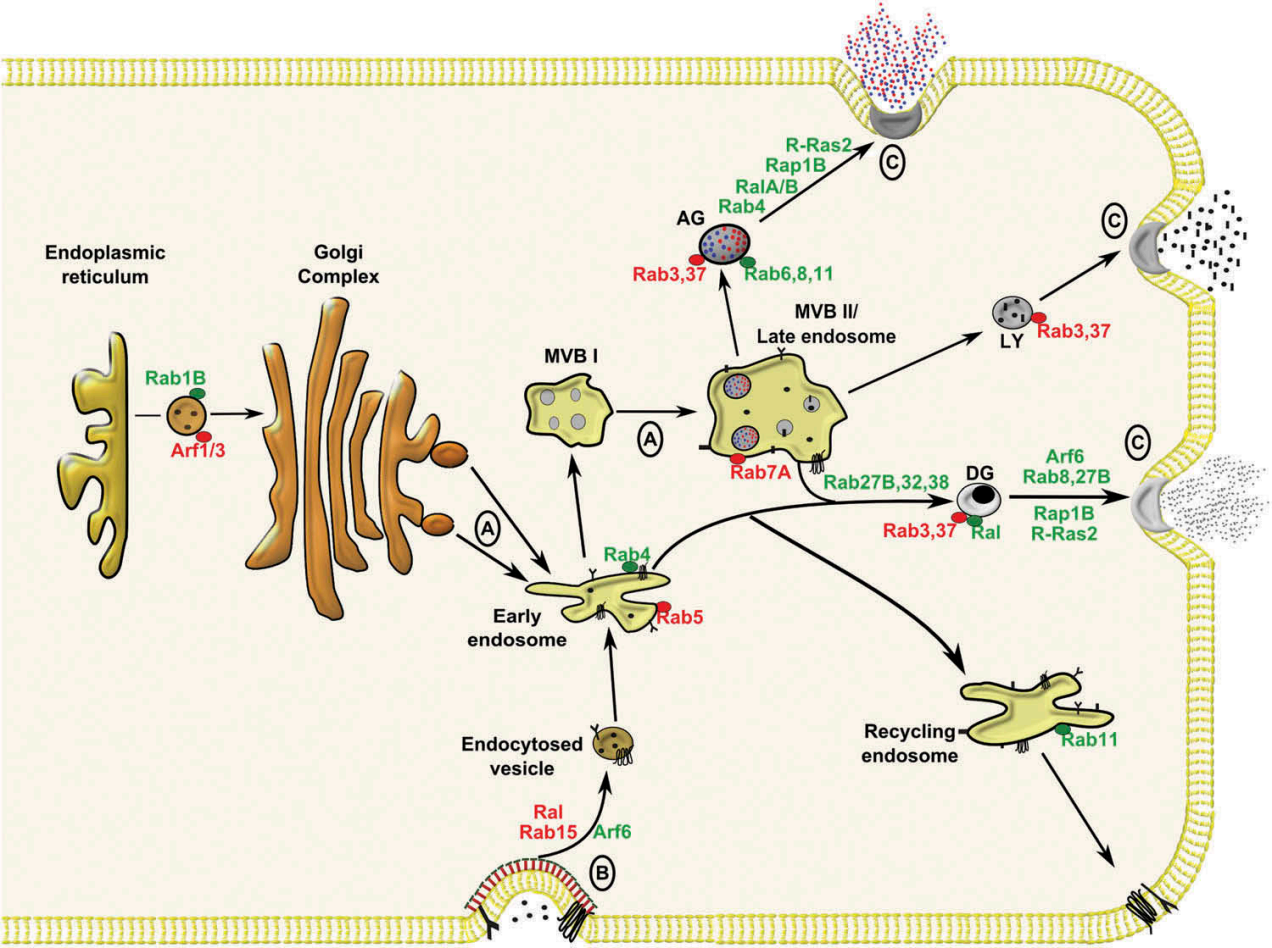
Membrane trafficking events within megakaryocytes and platelets involving Rab, Arf and Ras GTPases. (A) Granule biogenesis within the megakaryocyte involves input from biosynthetic and (B) endocytic pathways culminating in cargo packaging into early endosomes before sorting in immature multi-vesicular bodies (MVB I) to mature MVB II/late endosomes, or targeting to recycling endosomes. Following biogenesis, α-granules (AG), lysosomes (LY) and dense granules (DG) mature within platelets before targeting to the plasma membrane for (C) exocytosis. Trafficking roles for GTPases studied in megakaryocytes/platelets are highlighted in green, while roles for GTPases inferred from other cell systems are highlighted in red.

Platelet activity is no exception to these fundamental trafficking processes since they are critically reliant on exocytosis (secretion) and endocytosis to function effectively. Furthermore, trafficking is essential for vesicle (granule) biogenesis and maturation, which occurs within megakaryocytes (platelet precursors) and platelets, respectively (Figure 1) [3]. Secretion of distinctive cargo from platelets into blood occurs from three secretory granules, α-, dense and lysosomes. Secreted cargo is essential for limiting blood loss following acute/traumatic vessel injury (haemostasis), but can also exacerbate platelet activity in diseased blood vessels, causing occlusive ischaemic tissue damage (thrombosis) [4]. Dense granules (~ 3–8/platelet), are loaded with small signalling elements (ADP, ATP, 5HT, Ca^2+^, Mg^2+^ and polyphosphates), which act as autocrine/paracrine agents to amplify platelet activation [5]. Lysosomes (~ 1–3/platelet) are the least well-studied of platelet granules, but it is accepted that they secrete proteolytic enzymes (cathepsins, β-hexosaminidase) involved in clot remodelling processes [6]. On the other hand, platelet α-granules (~ 50–80/platelet) have been the subject of intensive investigation, mostly notably because of the vast array of cargo (chemokines, coagulation factors, adhesive proteins, antimicrobial peptides, growth factors) they release [7]. It is, therefore, unsurprising that platelets, through secreted cargo, also exhibit functions beyond haemostasis, such as inflammation, angiogenesis and tissue repair [8–10]. This in turn has led to a thorough investigation of the secretory machinery regulating platelet secretion. In particular, the molecular mechanisms of granule fusion with the plasma membrane are well described and require a number of key Soluble N-ethylmaleimide-sensitive factor Attachment protein REceptor (SNARE) proteins, including vesicle associated membrane protein (VAMP)-8, synaptosome associated protein (SNAP)-23 and syntaxin 11 and regulatory proteins such as Munc18b, Munc13-4 and syntaxin binding protein 5 (STXBP5) [11,12].

On the contrary, mechanistic insight into endocytic pathways in platelets are less clearly defined. Interestingly, the concept of endocytosis in platelets was first reported almost 30 years ago, but it has only been in recent years that studies have suggested more critical, functional roles for endocytosis in platelets [13,14]. We have previously reported endocytosis and trafficking of G-protein coupled receptors in platelets via clathrin-dependent pathways [15]. Our understanding of platelet granule biogenesis has been largely inferred from studies on human patients with granule deficiencies (referred to as storage pool deficiency) and related murine models [3]. It is believed that α-granules are derived from the biosynthetic and endocytic pathways, where cargoes are initially packaged within early endosomes before targeting to multi-vesicular bodies (MVBs)/late endosomes from which α-granules mature (Figure 1). The mechanism of dense granule biogenesis is more speculative, but is believed to require input from early endosomes, which may mature in MVBs. It is at this point, that the different granule types appear to mature via distinct machinery. For instance, mutations in the *NBEAL2* gene cause grey platelet syndrome (GPS), a disorder characterised by α-granule biogenesis defects, but patients have normal dense granule numbers. Similarly patients with Hermansky-Pudlack syndrome (HPS), have deficiencies in dense granules, but not α-granules [16]. Platelet granule biogenesis, has been the subject of recent review articles where greater detail can be found about this process [3,17].

The purpose of this review was to explore the roles of the Rab, Arf and Ras families of small guanosine triphosphatases (GTPases) with respect to platelet membrane trafficking events (exocytosis, endocytosis and granule biogenesis) and provide speculative arguments on trafficking roles that other platelet expressed GTPases may assume. Categorised on the basis of sequence homology, these three families of GTPases comprise over 130 proteins, which function as molecular switches cycling between an inactive ‘GDP-bound’ form to an active ‘GTP-bound’ form to regulate specific effector proteins which can also function beyond membrane trafficking events. The GTPase activities of these proteins are regulated by two classes of proteins; guanine nucleotide exchange factors (GEFs) which promote the GTP-bound form, whereas GTPase-activating proteins (GAPs) accelerate GTP hydrolysis to inactivate the GTPase [18]. Another key feature of these GTPases are the membrane-targeting sequences within the C-termini of Rab and Ras GTPases and N-termini of Arf GTPases, which control their membrane association and biological activity through lipid modifications [19]. We acknowledge there are other important regulators of vesicle trafficking in platelets, such as cytoskeletal proteins, kinases such as PKCα and PKD2, the large GTPase dynamin and dynamin-related protein 1 that are not discussed here, but we refer the reader to a number of relevant publications [20–25]. We also recommend a recent review on RhoGTPases in platelets, which details roles for specific Rho family members with respect to platelet secretion [26].

## The Rab GTPase Family in Platelets

**Figure 2.**
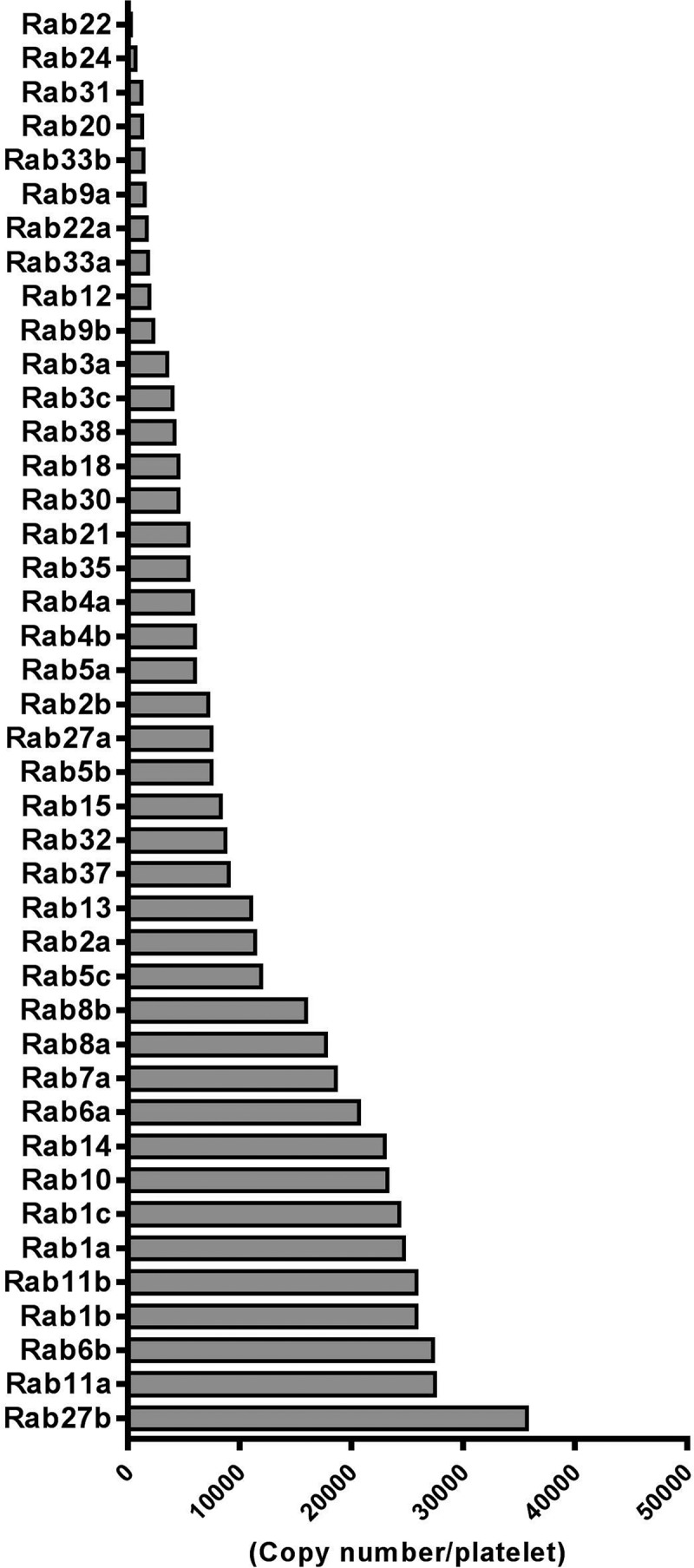
Ranked protein expression of 42 Rab GTPases in human platelets as detected in the Burkhart platelet proteome screen [28].

Initially described as Ras-like proteins in the brain (Rab), Rab GTPases make up the most abundant family of small GTPases, comprising almost 70 human members and are often described as master regulators of vesicle trafficking within endocytic and exocytotic pathways [27]. As a result, Rabs provide specific membrane identity, whilst ensuring that membrane-enclosed cargoes are trafficked to the correct cellular compartments. Based on proteomic studies, human platelets contain over 40 Rab members, with a range of expression levels from highly expressed Rab27B (35,000 copies/platelet) to lowly expressed Rab22 (500 copies/platelet, Figure 2) [28]. A seminal paper by Karniguian et al. in 1993 first described the expression of Rabs 1,3,4,6 and 8 in human platelets, with Rabs 3,4 and 8 being phosphorylated in response to platelet activation, but the relevance of this modification to platelet vesicle trafficking is unknown [29]. Rabs exist in both soluble and membrane-associated forms, the latter being principally mediated by post-translational modification of geranylgeranylation. This modification to cysteine residues within the C-terminus of the GTPases is catalysed by Rab geranylgeranyl transferase (RGGT), which coordinates with Rab escort proteins (REPs) to guide targeting of the Rab protein to vesicle or plasma membranes [27]. Notably, a naturally occurring mutation in *gunmetal* mice, which causes a 75% reduction in RGGT activity due to an autosomal recessive mutation, leads to a restricted mouse phenotype causing thrombocytopenia, loss of platelet granule content (both α- and dense granules) and prolonged bleeding times [30,31]. GTP-activated Rabs can specifically interact with numerous effectors, including vesicle tethering and fusion regulators, adaptors, motor proteins, kinases and phosphatases to regulate specific trafficking events [32]. Despite the high abundance of different Rab family members, there are limited functional studies on these GTPases in platelets, which likely reflects the limited availability of reliable pharmacological or molecular tools to target them within the anucleate platelet.

Studies in *ashen* mice, which lack Rab27A, suggested this Rab GTPase member was important for dense granule secretion and biogenesis, but it was subsequently shown that the genetic background of these mice was responsible for the granule defect [33,34]. Further work by Tolmachova et al. clearly demonstrated that platelets from Rab27B knockout mice (KO) exhibit a 50% reduction in dense granule numbers and impaired dense, but not α-, granule cargo release [35]. Notably, double KO (Rab27A/B^−/-^) platelets displayed slightly exaggerated dense granule release defects suggesting partial redundancy between isoforms, while reduction of dense granule numbers in Rab27B KO platelets was believed to reflect a packaging defect during megakaryocyte maturation [35]. Griscelli syndrome (GS) is one of the earliest examples of mutations in a Rab gene specifically implicated in human disease, where defects in the *RAB27A* gene cause partial albinism and hemophagocytic syndrome, characterised by uncontrolled T-cell and macrophage activation due to loss of cytolytic granule secretion [36]. No bleeding defects have been reported in type I GS patients, which is presumably due to compensation by the expression of Rab27B, as described above [1]. GTP-loaded Rab27B (and Rab27A) have been shown to directly interact with the SNARE tethering factor Munc13-4 in platelets, mutations of which cause familial hemophagocytic lymphohistiocytosis (FHL) 3, characterised by loss of lymphocyte cytotoxicity required for immune defence [37]. In these patients, secretion from lytic granules of cytotoxic T cells and natural killer cells is blocked, as these granules fail to properly ‘fuse’ with the plasma membrane [38]. Interestingly, platelets from FHL3 patients and Unc13d*^Jinx^* mice, which lack Munc13-4, have a complete ablation of dense granule secretion, suggesting regulators other than Rab27A/B are also important in facilitating dense granule secretion [39–41]. Also importantly and in contrast to the Rab27B knockout, Munc13-4 deficient platelets have normal granule numbers, suggesting other Rab27 effectors mediate granule packaging or biogenesis. Studies in melanosomes have shown the actin motor protein, myosin Va, can interact with Rab27 to regulate vesicle motility, but mouse platelets lacking myosin Va have no secretion defects [42].

In addition to Rab27 isoforms, Rab8 has been shown to regulate dense granule secretion, through interaction with the tethering factor synaptotagamin-like protein (SLP) 4, suggesting a highly complex and coordinated input from different Rab GTPases to facilitate dense granule release [43]. In the Karniguian paper, Rab8 and Rab6, were also found associated with platelet α-granules, while a study using a permeabilised platelet system showed that calcium-induced exocytosis of vWF from α-granules requires Rab4 [29,44]. Rab3B directly associated with the calcium binding protein calmodulin, which predominantly localised to the particulate fraction of human platelets, suggesting an association of this GTPase with platelet granules and/or the plasma membrane [45]. In other cell systems, Rab3, Rab26 and Rab37 are found associated with exocytotic vesicles, and based on proteomic expression in platelets (Figure 2), it is, therefore, possible that Rab37 may also have an important role in platelet secretion [27]. Interestingly, a recent study in mast cells identified Rab37 as a negative regulator of degranulation, which formed a complex with Munc13-4 and Rab27 and obscured vesicle docking/priming activity [46]. It would, therefore, be interesting to assess if such a role is assumed by Rab37 in platelets.

Mechanistic insights into endocytic pathways in platelets have also been uncovered. A study by Banerjee et al. revealed that VAMP3 regulates receptor-mediated endocytosis of fibrinogen and transferrin, most likely via a clathrin-dependent mechanism [47]. Notably, the authors also showed that fluid phase pinocytosis of labelled dextran into platelets was only marginally affected in VAMP3^−/-^ platelets, consistent with a previous report suggesting alternative endocytic pathways are present in platelets, but require distinct mechanisms [48]. Consistent with other mammalian cells, this internalised cargo was initially trafficked through Rab4 (early endosomes) positive compartments, before targeting to Rab11 (recycling endosomes) and vWF (α-granule) compartments. Beyond platelets, Rab5 is another GTPase specifically localised to early endosomes that regulates endocytosis and it has been recently reported that Rab5 isoforms (A, B and C) differentially regulate distinct endocytic pathways, presumably through interaction with different effectors [49]. These three isoforms are relatively abundant in human (and mouse) platelets and might be suggestive of a node through which internalised material is differentially trafficked in platelets towards recycling or degrading lysosomal compartments (Figure 2). Similarly, a study in CHO cells showed that Rab15 differentially regulates early steps in endocytic trafficking [50]. Regarding ‘later’ stages in endosomal vesicle trafficking, Rab7A is often associated with late endocytic compartments and transport to lysosomes. Rab7B controls endosomal transport to the Golgi complex, but proteomic data only supports expression of Rab7A in platelets (Figure 2) [51]. Additionally, the presence of a Golgi structure in platelets has been recently called into question, as Yadav et al. could only confirm the presence of scattered Golgi proteins, existing as separate structures whose function remained to be determined [52].

In megakaryocytes, downregulation of Rab1B, through loss/haplodeficiency of the transcription factor, RUNX1, alters early vesicle transport from the endoplasmic reticulum (ER) to the Golgi complex causing a reduction in content of the α-granule protein von Willebrand factor (vWF) [53]. Furthermore, Ninkovic et al. showed that fawn-hooded hypertensive rats which lack Rab38, due to a point mutation in the gene start codon, have no apparent dense granules within megakaryocytes or platelets [54]. A subsequent study in cultured MEG-O1 cells showed that Rab32 and Rab38 non-redundantly regulate dense granule biogenesis and both Rab GTPases were shown to specifically traffic dense granule cargo-containing vesicles to immature, Rab7A positive late endosomal/MVBs [55]. As mentioned, loss of dense granules is a classical feature of HPS, in which patients present with numerous clinical manifestations, including a bleeding diathesis due to loss of secreted ADP [56]. Mutations in 10 human genes are associated with HPS, including the Rab GEFs, HPS1 and HPS4, which specifically regulate Rab32 and Rab 38 activity, thereby highlighting a possible role for Rab GEFs in granule biogenesis [57]. Other regulators of Rab GTPase activity present in platelets include the GEFs; Rab3A-interacting protein (regulates Rabs8A and 8B), Rabex-5 (regulates Rab5a), vacuole protein sorting (VPS) 39-like protein (regulates Rab7) and GAPs; RabGAP1 (regulates Rab6), RabGAP1-like (regulates Rab22A) and Rab3GAP1 (regulates all Rab3 isoforms), but there is currently no evidence supporting their functional relevance to trafficking events in platelets [28]. Many questions remain for the field of Rab GTPases in platelet function and membrane trafficking. For instance, is internalised cargo trafficked to α-granules, targeted for lysosomal degradation or simply ‘lost’ within the Golgi or other endosomal compartments in platelets? It would also be interesting to assess the trafficking roles other highly expressed Rab GTPases in platelets, such as Rab10 and Rab14, but at present there is a lack of genetic or pharmacological data to speculate upon these roles (Figure 2).

## The Arf GTPase Family in Platelets

**Figure 3.**
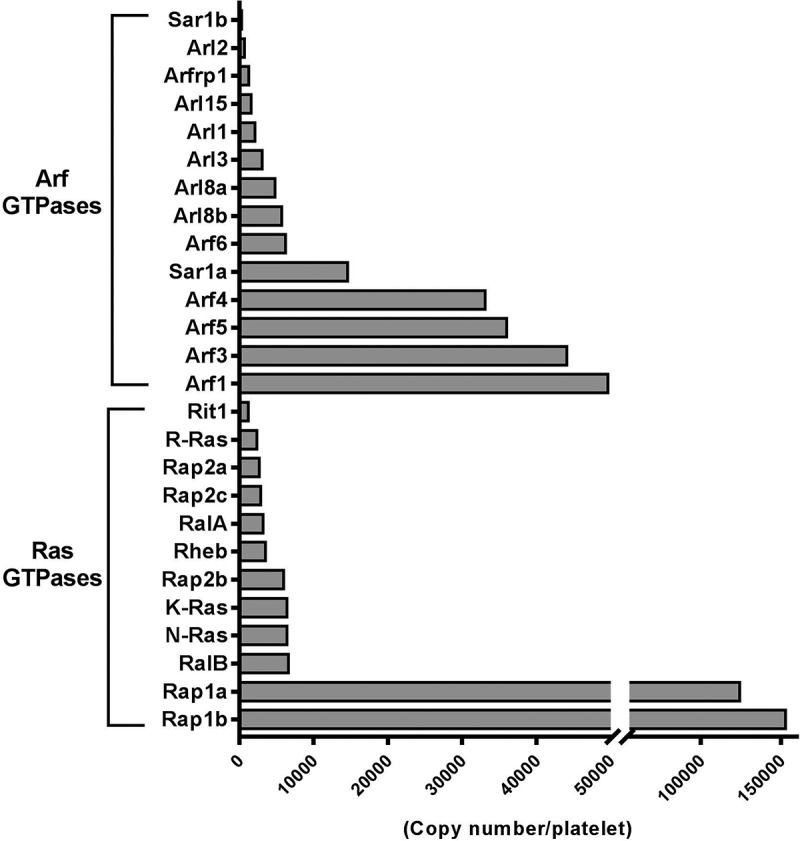
Ranked protein expression of 14 Arf GTPases and 12 Ras GTPases in human platelets as detected in the Burkhart platelet proteome screen [28].

The ADP-ribosylation factor (Arf) family of small GTPases comprises six founding members that are separated into three classes largely based on sequence homology; Class I (Arfs 1–3), Class II (Arfs 4–5) and Class III (Arf6). It is now recognised that these Arf proteins form part of a larger family containing over 20 proteins consisting of Arf-like (Arl), Arf-related (Arfrp) and the distally related, secretion-associated and Ras-related (Sar) proteins [58]. Figure 3 details the expression profile for this family in platelets, with the founding Arf members being the most highly expressed and a total of 14 Arf family members have been identified. Similar to Rab GTPases, Arfs are under tight spatial and functional control by GEFs and GAPs, but a distinguishing feature is the myristoylation at the N terminus which brings the GTPase in close contact with the membrane allowing for biological activity [59]. Arf effectors, which include coat proteins involved in endocytosis, membrane tethers, lipid enzymes and scaffold proteins, preferentially bind the GTP-bound form of Arfs, but the GDP-bound form can also interact with separate targets to regulate trafficking events [60]. Beyond trafficking-associated functions, the Arf family also plays important roles in cytoskeletal remodelling, cytokinesis and lipid droplet formation [60,61]. Various human diseases with mutations in Arf family proteins have begun to emerge, but to the best of our knowledge there are currently no reports of associated bleeding diathesis.

Of the six Arf proteins, Arf6 has received the most attention for its roles in platelets. Choi et al. first demonstrated that Arf6 is a key element in platelet activation through GPVI- and PAR-mediated signalling [62,63]. Interestingly, Arf6-GTP was the predominant form in resting platelets and the level of this GTP-bound state rapidly decreased upon stimulation of platelets with collagen and convulxin, which is unusual since GTPases more commonly exist in inactive GDP-bound states in resting cells. It is possible that under resting conditions, platelets are undergoing active trafficking events involving Arf6, which is favourably localised to the plasma membrane. Indeed, Arf6-GTP-mediated regulation of phospholipase D and phosphatidylinositol 4-phosphate 5-kinase facilitates structural changes in the plasma membrane, allowing recruitment of coat proteins involved in clathrin-mediated endocytosis [64]. Kanamarlapudi et al. later showed that Arf6 activity was also important for P2Y purinoceptor internalisation and subsequent trafficking events which are critical for the resensitisation and function of these receptors in platelets [65]. Conditional gene knockout of Arf6 in mouse platelets resulted in only a mild platelet phenotype due to a defect in fibrinogen uptake by platelets through integrin α_IIb_β_3_-mediated endocytic trafficking [66]. Deletion of Arf6 specifically enhanced platelet spreading on fibrinogen and accelerated clot retraction, but did not affect murine platelet aggregation, surface α_IIb_β_3_ activation or in vivo thrombosis. The mechanism underlying the enhanced platelet spreading however was not completely understood and was speculated to result from more rapid recycling of activated α_IIb_β_3_ to and from the surface of Arf6 null platelets [66]. Notably, the earlier study by Choi *et al*. in human platelets reported defective platelet aggregation with a myristoylated (myr)-Arf6 peptide, which preserved Arf6-GTP upon platelet activation [62]. Importantly, the myr-Arf6 peptide also inhibited fibrinogen endocytosis in human platelets, but it not clear if the functional differences between human and mouse platelets is caused by off-target effects of the myr-Arf6 peptide, or due to fundamental differences between species [66].

Besides Arf6, Arf1 and Arf3 represent the most studied Arf members in other cell systems and are more abundant in platelets than Arf6, sharing 96% sequence identity (Figure 3). Most evidence to date supports identical roles for these GTPases in vesicle transport along the biosynthetic pathway, between the endoplasmic reticulum and Golgi compartments, by recruiting coated vesicles allowing for cargo selection and packaging [61]. It is tempting to speculate critical roles for these Arfs along the biosynthetic pathway of α-granule biogenesis within megakaryocytes, but also for their maturation within platelets. Similar roles have been reported for Sar1, of which the Sar1A isoform is relatively abundant in human platelets [67]. In addition, numerous Arf regulators are present in platelets including the GEFs; GBF1, BIG2, CYH2 and GAPs; ArfGAP1/2, ASAP1/2, GIT1/2 and ARAP1, some of which were found complexed within a phosphatidylinositol triphosphate interactome at the plasma membrane of platelets to coordinate signalling events [28,68]. Notably, work by our group demonstrated that Arf6 also controls exocytic pathways in resting platelets, by restraining dense granule secretion via a direct interaction with the Arf GEF, CYH2 (cytohesin-2), which maintained Arf6 in a GTP-bound state. Following activation, CYH2 was phosphorylated by PKC, leading to a dissociation from and inactivation of Arf6, which facilitated granule secretion [69]. In addition to GEFs/GAPs, protein interactions are likely to regulate Arf activity. One piece of evidence comes from Urban and colleagues who showed that deletion of Pdlim7, a PDZ-LIM family protein, resulted in unbalanced Arf6-GDP/GTP ratios in mice, although the mechanism behind this is not clear [70]. Interestingly, perinatal lethality due to spontaneous systemic thrombosis was reported in these mice. However, mice lacking platelet Arf6 have no reported incidence of spontaneous thrombi suggesting the phenotype in Pdlim7 deficient mice is likely independent of Arf6. Undoubtedly, much further experimental work is required to fully understand the functional roles and regulation of Arfs with respect to trafficking events in platelets.

## The Ras GTPase Family in Platelets

The Ras sarcoma (Ras) GTPases are the second largest family of the Ras superfamily containing 36 members classically associated with cell proliferation, differentiation and survival, while approximately 30% of all human cancers contain Ras activating mutations [71]. Numerous Ras members are expressed in platelets (Figure 3) and early studies demonstrated that Ras could be activated following platelet stimulation [72,73]. However this did not result in the classical activation of the MAP kinase ERK, as is the case in nucleated cells and the relevance of K-, N- and R-Ras GTPases in platelet function remains unclear. Notably, mutations in four different genes associated with Ras signalling, including *K-RAS*, are linked to the autosomal dominant disorder, Noonan syndrome (NS) [74]. These patients present with a bleeding diathesis and defects in platelet function have been reported, although *K-RAS* mutations only account for < 2% of NS patients [75,76]. Interestingly, a recent publication by Janapati et al. demonstrated a role for R-Ras2 (TC21) in GPVI-induced integrin activation, granule secretion and thrombus formation, which acted upstream of Rap1B [77]. Rap1 (A and B) are the most abundantly expressed Ras members in platelets and critical roles with respect to platelet integrin activation and adhesiveness have been established, while roles for the much lower expressed Rap2 (A, B and C) members remain elusive. Zhang and colleagues demonstrated that Rap1B is also important for granule (α- and dense) secretion, although the underlying mechanism is not known [78]. For further information on Rap signalling in platelets, we refer the reader to a recent review by Stefanini and Bergmeier [79].

Of particular relevance to this review are the Ras-like (Ral) GTPases, RalA and RalB. Like most Ras GTPases, Rals have been implicated in numerous cellular processes including oncogenic transformations, but have also been implicated as regulators of vesicle trafficking [80]. They are ubiquitously expressed, with high abundance in brain, testes and platelets sharing 82% sequence identity, with ~ 55% identity to other Ras GTPases [81]. A well-characterised effector of Rals is the exocyst complex, comprising of 8 proteins (EXOC1-8) that facilitate the tethering of exocytic vesicles to the plasma membrane [82]. Notably, all components of this octameric complex are present in human (and mouse) platelets and GTP-loaded Rals have been shown to bind EXOC2 and EXOC8 in a mutually exclusive manner [83]. Similarly, other established effectors of Rals include Ral binding protein (RalBP1) and phospholipase D, but their relevance to platelet function and/or vesicle trafficking remains to be determined. Of relevance, RalBP1 has been shown to play a role in receptor-mediated endocytosis by interacting with epsin homology domain proteins Reps1 and Reps2, aswell as clathrin adaptor protein complex AP2, all of which are present in platelets and are supportive of an endocytic function for platelet Rals (Figure 1) [84].

Within platelets, Ral was found associated with dense granules [85] and can be activated by various platelet agonists in a Ca^2+^-dependent manner, while studies in endothelial cells support a role for RalA in Weibel Palade body exocytosis [86,87]. Interestingly, it is well characterised that Ral activity is regulated by numerous Ras GTPases, including H-Ras, R-Ras, Rap 1 (A and B), which directly bind to and regulate Ral GEFs, including RalGDS and RGL (1–3) [88]. Proteomic data analysis only supports the expression of RGL2 in murine platelets, while the RalGAP α1, α2 and β subunits are present in human (and mouse) platelets to regulate Ral-GTP hydrolysis [28,89]. Considering the importance of Rap to platelet secretion, if would be interesting to assess a potential crosstalk between Rap and Ral GTPases. Functional studies involving permeabilised human platelets alluded to a role for Rals in dense granule secretion, through an interaction with EXOC2 [90]. However, a recent study from our group demonstrated that murine platelets lacking either RalA or RalB have no defect in dense granule cargo release, while RalA/B double KO (DKO) platelets have only a marginal defect which does not alter functional responses such as aggregation and thrombus formation [91]. Interestingly, platelet surface exposure of P-selectin in DKO platelets was substantially reduced (by ~ 85%) in response to GPVI-mediated platelet activation, whereas the release of an array of soluble α-granule cargo was unaffected. Notably, this exocytic defect in α-granule release of P-selectin was not due to the minor reduction in secreted ADP from dense granules. This also reveals mutually redundant trafficking roles for both Ral proteins in platelets, which corroborates their roles in development and tumorigenesis [80]. These findings raise intriguing questions regarding exocytic trafficking processes in platelets. Firstly, do ‘kiss-and-run’ secretion events occur, where soluble cargo are readily released upon transient fusion of the secretory vesicle at the plasma membrane? An alternative explanation is the presence of P-selectin positive and negative α-granule subpopulations, as it has been reported to be differentially localised to another α-granule protein, vWF, and thereby Rals could specifically target the P-selectin positive population [92]. Undoubtedly many questions remain regarding theories of cargo storage in platelets and mechanisms controlling their trafficking and release, but these observations reported in Ral deficient platelets offer attractive therapeutic potential [93,94].

## Conclusion

It has become increasingly apparent that small GTPases play pivotal roles in the regulation of membrane vesicle trafficking in anucleate platelets as they do in nuclear cells. They shuttle between cytosolic and membrane compartments, and as such they are well positioned to control these critical cellular processes. Our knowledge of these binary molecular switches in platelets has accumulated in the last two decades, particularly through research with genetic manipulation in animal models (see Table I). Furthermore, platelet proteomic datasets have provided key expression data on an array of small GTPases, whose relevance to platelet function remains to be determined. Crucially, these discoveries may led to the identification of specific targets that could pave the way for manipulating exocytic and endocytic processes in platelets. For instance, the ability to selectively modulate P-selectin mediated platelet-leukocyte interactions during inflammatory pathologies, without affecting the haemostatic properties of platelets. Reciprocally, it could be possible to selectively control the endocytic uptake of plasma proteins by platelets that could be useful for exploiting tissue regenerative roles for platelets, but also to control the sequestration of tumor proteins by platelets that primes distal sites, such as bone, for metastasis [95].

**Table I.** Summary of small GTPases discussed in this review. Mouse models are described, unless otherwise stated. Studies using pharmacological inhibitors or permeabilised platelet systems are not included.

		Effect on granule count/content/secretion				
Protein	Gene	Alpha	Dense	Lysosome	Model	Reference
Rab27A	*RAB27A*	Normal vWF levels, normal P-selectin exposure	Normal count/5HT content	nd	*Ashen* mouse *(Rab27a^−/-^* global)	Barral *et al*. (2002)
Rab27B	*RAB27B*	Normal P-selectin exposure	Reduced count/5HT content, secretion defect-further impaired in Rab27a/b null	nd	*Rab27b^−/-^* (global)	Tolmachova *et al*. (2007)
Rab38	*RAB38*	Normal count, and P-selectin exposure	None detected in Megs or Platelets	Normal count	FHH rat (*Rab38^−/-^* due to point mutation in gene ATG start site)	Ninkovic *et al*. (2008)
Rab32	*RAB32*	nd	Biogenesis defect in Megs	nd	*Rab32* siRNA (MEG-01 cells)	Ambrosio *et al*. (2012)
Rab38	*RAB38*	nd	Biogenesis defect in Megs	nd	*Rab38* siRNA (MEG-01 cells)	Ambrosio *et al*. (2012)
Arf6	*ARF6*	Normal count, decreased fibrinogen content	Normal count and secretion	nd	*Arf6^flox/flox^- PF4-Cre*	Huang *et al*. (2016)
RalA	*RALA*	Normal count, and secretion*	Normal count and secretion†	Normal count and secretion	*RalA ^flox/flox^-PF4-Cre*	Wersäll *et al*. (2018)
RalB	*RALB*	Normal count, P-selectin exposure defect*	Normal count and secretion†	Normal count and secretion	*RalB^flox/flox^- PF4-Cre*	Wersäll *et al*. (2018)
R-Ras2	*RRAS2*	GPVI-specific secretion defect	GPVI-specific secretion defect	nd	*Rras2^−/-^* (global)	Janapati *et al*. (2018)

‘*’ P-selectin exposure defect markedly enhanced and ‘†’ mild ATP secretion defect in RalA/B double knockout platelets, ‘nd’ indicates no data available, ‘FHH’ indicates fawn-hooded hypertensive.
